# 2-Amino-4-[1-(2-chloro­phen­yl)-5-methyl-1*H*-1,2,3-triazol-4-yl]-6-(4-methyl­phen­yl)benzene-1,3-dicarbonitrile

**DOI:** 10.1107/S1600536809014032

**Published:** 2009-04-25

**Authors:** Wang-Jun Dong, Hui-Cheng Wang, Zhong-Liang Gao, Rong-Shan Li, Heng-Shan Dong

**Affiliations:** a State Key Laboratory of Applied Organic Chemistry, Institute of Organic Chemistry, College of Chemistry and Chemical Engineering, Lanzhou University, Gansu 730000, People’s Republic of China

## Abstract

In the title compound, C_24_H_17_ClN_6_, the dihedral angles between the triazolyl ring and its adjacent chlorobenzene and trisubstituted benzene rings are 90.6 (2) and 55.7 (3)°, respectively. The dihedral angle between the trisubstituted ring and the attached tolyl ring of the biphenyl unit is 45.9 (3)°. Intra- and intermolecular N—H⋯N hydrogen bonds are present.

## Related literature

For the synthesis, see: Victory *et al.* (1991 ▶).
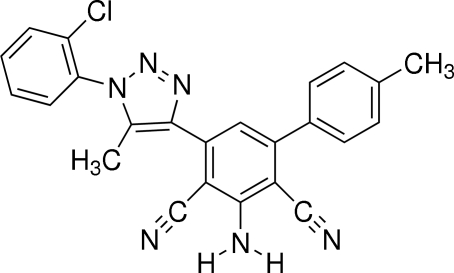


## Experimental

### 

#### Crystal data

C_24_H_17_ClN_6_
*M* *_r_* = 424.89
Monoclinic, 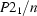

*a* = 13.623 (6) Å
*b* = 7.792 (4) Å
*c* = 20.608 (10) Å
β = 103.502 (6)°
*V* = 2127.0 (17) Å^3^
*Z* = 4
Mo *K*α radiation
μ = 0.20 mm^−1^
*T* = 293 K
0.33 × 0.31 × 0.29 mm

#### Data collection

Bruker APEXII diffractometer
Absorption correction: multi-scan (*SADABS*; Sheldrick, 1996 ▶) *T*_min_ = 0.935, *T*_max_ = 0.943
11092 measured reflections
4169 independent reflections
3263 reflections with *I* > 2σ(*I*)
*R*_int_ = 0.020

#### Refinement

*R*[*F* ^2^ > 2σ(*F* ^2^)] = 0.043
*wR*(*F* ^2^) = 0.120
*S* = 1.03
4169 reflections
282 parameters
H-atom parameters constrained
Δρ_max_ = 0.24 e Å^−3^
Δρ_min_ = −0.33 e Å^−3^

### 

Data collection: *APEX2* (Bruker, 2004 ▶); cell refinement: *SAINT* (Bruker, 2004 ▶); data reduction: *SAINT*; program(s) used to solve structure: *SHELXS97* (Sheldrick, 2008 ▶); program(s) used to refine structure: *SHELXL97* (Sheldrick, 2008 ▶); molecular graphics: *PLATON* (Spek, 2009 ▶); software used to prepare material for publication: *publCIF* (Westrip, 2009 ▶).

## Supplementary Material

Crystal structure: contains datablocks I, global. DOI: 10.1107/S1600536809014032/ng2572sup1.cif

Structure factors: contains datablocks I. DOI: 10.1107/S1600536809014032/ng2572Isup2.hkl

Additional supplementary materials:  crystallographic information; 3D view; checkCIF report

## Figures and Tables

**Table 1:** Hydrogen-bond geometry (Å, °)

*D*—H⋯*A*	*D*—H	H⋯*A*	*D*⋯*A*	*D*—H⋯*A*
N5—H5*B*⋯N6^i^	0.86	2.56	3.221 (2)	134
N5—H5*A*⋯N4	0.86	2.91	3.540 (2)	125

Symmetry code: (i) 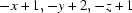.

**Fig. 1.**
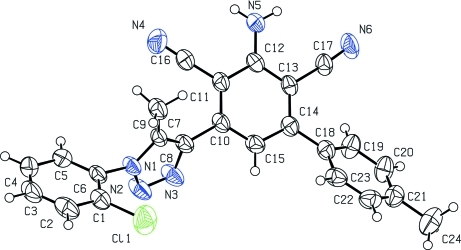
A PLATON (Spek, 2009) view of the molecular structure of (I). The asymmetric unit showing 50% probability displacement ellipsoids.

**Fig. 2.**
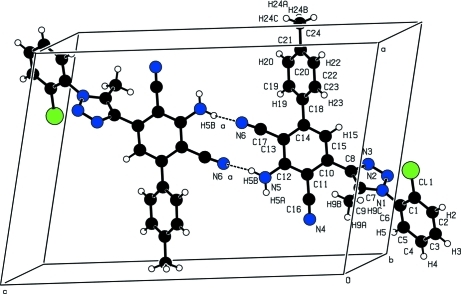
A PLATON (Spek, 2009) view of the hydrogen-bonded motif of the supramolecular structure. Hydrogen bonds are shown as dashed lines. [Symmetry codes: (i) -x + 1/2, y - 1/2, -z + 1/2.]

## supplementary crystallographic information

### Comment

The title compound, C_24_H_17_N_6_Cl (I) (Figuer 1) was synthesized by the reaction of (*E*)-1-[1-(2-chlorophenyl)-5-methyl-1*H*-1,2,3-triazol-4-yl]-3-(4-methylphenyl)prop-2-en-1-ones and malononitrile in the presence of piperindine at 25°C. The consists plane of subsituted triazolyl ring and 2-chlorophenyl ring, subsituted triazolyl ring and 2-amino-1,3-dinitrilphenyl ring, 2-amino-1,3-dinitrilphenyl ring and 4-methylphenyl ring is not co-planar [The dihedral angle of C1—C6—N1—N2 is 90.6 (2)°, C7—C8—C10—C11 is 55.7 (3)°, C13—C14—C18—C19 is 45.9 (3)° in stable conformation of the crystal].

On 2-amino-1,3-dinitrilphenyl ring, the *p*-π conjugation was indicated the between amino N5 and ring C12, bond length of C12—N5 is 1.361 (2)Å which is shorter than non-conjugation Csp2-Nsp2 bond C6—N1 1.430 Å(Table 1), angle of C12—N5—H5A, H5A—N5—H5B is 120°, the dihedral angle of N5—C12—C13—C14 is 177.3°, the dihedral angle of N5—C12—C13—C17 is 0.9°, N5 is sp2 hybridized atom.

On 2-amino-1,3-dinitrilphenyl ring, 2-amino has two N—H bond, and two intermolecular hydrogen bonds as the supramolecular structure in the crystal. The intermolecular N6···H5B—N5 hydrogen bond between the N6 atoms of C≡N group and N5—H5B, intermolecular N6···H'5B-N'5 hydrogen bond between the N6 atoms of the C≡N group and N5-H5B (Figure 2; Table 2). One 12 members ring is consisted of two intermolecular H-bonds. The orderly range of the structure forms stratification polymer in the crystal. The intermolecular hydrogen bond connect the translated molecules into an infinite chain on a layer.

### Experimental

2-Amino-4-[1-(4-chlorophenyl)-5-methyl-1*H*-1,2,3-triazol-4-yl]-6-(4-methylphenyl)benzene-1,3-dinitrile, which was synthesized by a modification of a published procedure (Victory, *et al.* 1991). To add 1.6 mL piperidine the mixture liquor of 0.675 g (2 mmol) (*E*)-1-[1-(2-chlorophenyl)-5-methyl-1*H*-1,2,3-triazol-4-yl]-3-(4-methylphenyl)prop-2-en-1-one and 0.264 g (4 mmol) malononitrile in 5 mL of absolute ethanol was stirred. The mixture was stirred for 30 h at room temperature. After removal of solvent, the mixture was poured into water, neutrilized with 10% acetic acid. The resulting solid was filtered, washed with water, dried and isolated with petroleum ether/EtOAc(4:1) to give the target compound, mp 470–471 K, in 75% yield. The structure was established by ^1^H-NMR, IR and mass spectrosopic data analyses. ^1^H NMR(300 MHz, CDCl_3_): δ=2.341 (s, 3H, Ar—CH_3_), 2.458 (s, 3H, triazolyl-CH_3_), 5.408 (s, 2H, NH_2_), 7.161 (s, 1H, 5-H), 7.308–7.335 (d, 2H, *J *= 8.1 Hz, Ar—H), 7.485–7.601 (m, 5H, Ar—H), 7.636–7.663(d, 1H, *J *= 8.1 Hz, Ar—H) p.p.m.. MS (%): 424 (*M*^+.^, 1.35%), 396(19.8), 334(3.5), 320(2.6), 305(1.5), 256 (3.3), 232 (1.2), 205 (3.5), 192 (11.7), 164 (10.6), 178(4.5), 164(10.6), 141(6.9), 138(10.3), 125(10.3), 91(17.5), 85(25.3), 77(10.6), 57(51.4), 43(100.0). IR(KBr, cm^-1^): 3442, 3349, 3234(N—H), 2214(C≡N), 1634, 1551, 1495, 823, 766

### Refinement

Carbon-bound H-atoms were placed in calculated positions (C—H 0.93 to 0.98 Å) and were included in the refinement in the riding model approximation, with *U*(H) set to 1.2 to 1.5*U*_eq_(C). The methyl groups were rotated to fit the electron density.  The amino H-atoms were similarly generated.

### Crystal data

**Table:**

C_24_H_17_ClN_6_	*F*(000) = 880
*M**_r_* = 424.89	*D*_x_ = 1.327 Mg m^−^^3^
Monoclinic, *P*2_1_/*n*	Melting point: 470 K
Hall symbol: -p 2yn	Mo *K*α radiation, λ = 0.71073 Å
*a* = 13.623 (6) Å	Cell parameters from 4544 reflections
*b* = 7.792 (4) Å	θ = 2.6–29.3°
*c* = 20.608 (10) Å	µ = 0.20 mm^−^^1^
β = 103.502 (6)°	*T* = 293 K
*V* = 2127.0 (17) Å^3^	Block, colorless
*Z* = 4	0.33 × 0.31 × 0.29 mm

### Data collection

**Table:**

Bruker APEXII diffractometer	4169 independent reflections
Radiation source: fine-focus sealed tube	3263 reflections with *I* > 2σ(*I*)
graphite	*R*_int_ = 0.020
φ and ω scans	θ_max_ = 26.0°, θ_min_ = 1.6°
Absorption correction: multi-scan (*SADABS*; Sheldrick, 1996)	*h* = −16→14
*T*_min_ = 0.935, *T*_max_ = 0.943	*k* = −6→9
11092 measured reflections	*l* = −25→24

### Refinement

**Table:**

Refinement on *F*^2^	Primary atom site location: structure-invariant direct methods
Least-squares matrix: full	Secondary atom site location: difference Fourier map
*R*[*F*^2^ > 2σ(*F*^2^)] = 0.043	Hydrogen site location: inferred from neighbouring sites
*wR*(*F*^2^) = 0.120	H-atom parameters constrained
*S* = 1.03	*w* = 1/[s^2^(*F*_o_^2^) + (0.0573*P*)^2^ + 0.5879*P*] where *P* = (*F*_o_^2^ + 2*F*_c_^2^)/3
4169 reflections	(Δ/σ)_max_ < 0.001
282 parameters	Δρ_max_ = 0.24 e Å^−^^3^
0 restraints	Δρ_min_ = −0.33 e Å^−^^3^

### Special details

**Table:**

Geometry. All e.s.d.'s (except the e.s.d. in the dihedral angle between two l.s. planes) are estimated using the full covariance matrix. The cell e.s.d.'s are taken into account individually in the estimation of e.s.d.'s in distances, angles and torsion angles; correlations between e.s.d.'s in cell parameters are only used when they are defined by crystal symmetry. An approximate (isotropic) treatment of cell e.s.d.'s is used for estimating e.s.d.'s involving l.s. planes.
Refinement. Refinement of *F*^2^ against ALL reflections. The weighted *R*-factor *wR* and goodness of fit *S* are based on *F*^2^, conventional *R*-factors *R* are based on *F*, with *F* set to zero for negative *F*^2^. The threshold expression of *F*^2^ > σ(*F*^2^) is used only for calculating *R*-factors(gt) *etc*. and is not relevant to the choice of reflections for refinement. *R*-factors based on *F*^2^ are statistically about twice as large as those based on *F*, and *R*- factors based on ALL data will be even larger.

### Fractional atomic coordinates and isotropic or equivalent isotropic displacement parameters (Å^2^)

**Table:**

	*x*	*y*	*z*	*U*_iso_*/*U*_eq_	
C1	0.24428 (14)	0.9337 (2)	−0.06466 (9)	0.0506 (4)	
C2	0.18147 (18)	0.9038 (3)	−0.12685 (9)	0.0620 (5)	
H2	0.2062	0.8490	−0.1598	0.074*	
C3	0.08287 (17)	0.9551 (3)	−0.13950 (9)	0.0667 (6)	
H3	0.0409	0.9343	−0.1813	0.080*	
C4	0.04481 (15)	1.0363 (3)	−0.09206 (10)	0.0655 (6)	
H4	−0.0225	1.0703	−0.1016	0.079*	
C5	0.10651 (13)	1.0676 (3)	−0.02996 (9)	0.0530 (5)	
H5	0.0811	1.1230	0.0026	0.064*	
C6	0.20613 (13)	1.0164 (2)	−0.01645 (7)	0.0417 (4)	
C7	0.29203 (12)	0.9574 (2)	0.10288 (7)	0.0391 (4)	
C8	0.35736 (13)	1.0573 (2)	0.14774 (8)	0.0435 (4)	
C9	0.25130 (14)	0.7827 (2)	0.10582 (9)	0.0515 (4)	
H9A	0.1810	0.7897	0.1061	0.077*	
H9B	0.2875	0.7265	0.1457	0.077*	
H9C	0.2589	0.7185	0.0675	0.077*	
C10	0.41031 (13)	1.0236 (2)	0.21766 (7)	0.0423 (4)	
C11	0.35630 (12)	0.9787 (2)	0.26529 (8)	0.0409 (4)	
C12	0.40644 (13)	0.9537 (2)	0.33256 (7)	0.0399 (4)	
C13	0.51190 (12)	0.9773 (2)	0.34982 (7)	0.0400 (4)	
C14	0.56677 (13)	1.0206 (2)	0.30216 (8)	0.0418 (4)	
C15	0.51383 (13)	1.0430 (2)	0.23641 (8)	0.0455 (4)	
H15	0.5489	1.0718	0.2043	0.055*	
C16	0.24834 (15)	0.9704 (3)	0.24816 (8)	0.0518 (5)	
C17	0.56104 (13)	0.9498 (2)	0.41871 (8)	0.0468 (4)	
C18	0.67814 (12)	1.0387 (2)	0.31955 (8)	0.0424 (4)	
C19	0.72827 (13)	1.1296 (3)	0.37549 (9)	0.0526 (5)	
H19	0.6915	1.1798	0.4033	0.063*	
C20	0.83163 (14)	1.1464 (3)	0.39034 (10)	0.0580 (5)	
H20	0.8633	1.2092	0.4278	0.070*	
C21	0.88966 (13)	1.0726 (2)	0.35108 (10)	0.0524 (4)	
C22	0.83984 (14)	0.9792 (2)	0.29615 (9)	0.0528 (5)	
H22	0.8772	0.9255	0.2695	0.063*	
C23	0.73613 (14)	0.9637 (2)	0.27994 (8)	0.0493 (4)	
H23	0.7046	0.9024	0.2421	0.059*	
C24	1.00282 (15)	1.0947 (3)	0.36777 (14)	0.0788 (7)	
H24A	1.0198	1.2071	0.3861	0.118*	
H24B	1.0273	1.0815	0.3280	0.118*	
H24C	1.0334	1.0096	0.3999	0.118*	
Cl1	0.36899 (5)	0.87178 (10)	−0.04831 (3)	0.0865 (2)	
N1	0.27058 (11)	1.05500 (18)	0.04722 (6)	0.0436 (3)	
N2	0.31963 (13)	1.2076 (2)	0.05662 (7)	0.0609 (5)	
N3	0.37317 (13)	1.2074 (2)	0.11804 (7)	0.0608 (5)	
N4	0.16232 (13)	0.9651 (3)	0.23643 (9)	0.0800 (6)	
N5	0.35492 (11)	0.9051 (2)	0.37873 (6)	0.0488 (4)	
H5A	0.2907	0.8892	0.3670	0.059*	
H5B	0.3866	0.8903	0.4196	0.059*	
N6	0.59330 (13)	0.9292 (3)	0.47402 (7)	0.0686 (5)	

### Atomic displacement parameters (Å^2^)

**Table:**

	*U*^11^	*U*^22^	*U*^33^	*U*^12^	*U*^13^	*U*^23^
C1	0.0600 (11)	0.0520 (10)	0.0374 (9)	−0.0012 (9)	0.0063 (8)	0.0016 (8)
C2	0.0899 (16)	0.0608 (12)	0.0331 (9)	−0.0109 (11)	0.0102 (9)	−0.0041 (8)
C3	0.0724 (14)	0.0815 (15)	0.0353 (10)	−0.0277 (12)	−0.0096 (9)	0.0100 (10)
C4	0.0445 (10)	0.0927 (16)	0.0524 (12)	−0.0116 (10)	−0.0025 (9)	0.0216 (11)
C5	0.0468 (10)	0.0695 (12)	0.0425 (9)	−0.0044 (9)	0.0098 (8)	0.0101 (9)
C6	0.0493 (9)	0.0475 (9)	0.0249 (7)	−0.0064 (7)	0.0016 (6)	0.0054 (7)
C7	0.0415 (8)	0.0456 (9)	0.0268 (7)	−0.0027 (7)	0.0011 (6)	0.0033 (6)
C8	0.0495 (9)	0.0487 (10)	0.0289 (8)	−0.0089 (8)	0.0019 (7)	0.0025 (7)
C9	0.0573 (11)	0.0500 (10)	0.0398 (9)	−0.0106 (8)	−0.0036 (8)	0.0053 (8)
C10	0.0498 (10)	0.0443 (9)	0.0279 (8)	−0.0058 (7)	−0.0008 (7)	−0.0016 (7)
C11	0.0427 (9)	0.0444 (9)	0.0314 (8)	−0.0018 (7)	0.0001 (6)	−0.0007 (7)
C12	0.0493 (9)	0.0388 (9)	0.0294 (8)	0.0020 (7)	0.0046 (7)	−0.0013 (6)
C13	0.0451 (9)	0.0438 (9)	0.0271 (7)	0.0037 (7)	0.0004 (6)	−0.0033 (6)
C14	0.0457 (9)	0.0425 (9)	0.0336 (8)	−0.0032 (7)	0.0021 (7)	−0.0051 (7)
C15	0.0510 (10)	0.0528 (10)	0.0303 (8)	−0.0109 (8)	0.0049 (7)	−0.0004 (7)
C16	0.0518 (11)	0.0686 (12)	0.0312 (8)	−0.0008 (9)	0.0017 (7)	0.0015 (8)
C17	0.0439 (9)	0.0603 (11)	0.0333 (9)	0.0113 (8)	0.0033 (7)	−0.0053 (7)
C18	0.0448 (9)	0.0446 (9)	0.0350 (8)	−0.0016 (7)	0.0037 (7)	−0.0020 (7)
C19	0.0436 (10)	0.0603 (12)	0.0505 (10)	0.0035 (8)	0.0040 (8)	−0.0194 (9)
C20	0.0456 (10)	0.0610 (12)	0.0596 (11)	0.0017 (9)	−0.0032 (8)	−0.0177 (9)
C21	0.0439 (10)	0.0493 (10)	0.0624 (12)	0.0038 (8)	0.0089 (8)	0.0026 (9)
C22	0.0583 (11)	0.0524 (11)	0.0532 (11)	0.0038 (9)	0.0243 (9)	0.0020 (8)
C23	0.0596 (11)	0.0541 (11)	0.0348 (9)	−0.0081 (9)	0.0124 (8)	−0.0041 (7)
C24	0.0474 (12)	0.0752 (16)	0.1114 (19)	0.0027 (11)	0.0140 (12)	−0.0038 (14)
Cl1	0.0737 (4)	0.1036 (5)	0.0813 (4)	0.0322 (3)	0.0161 (3)	−0.0021 (3)
N1	0.0533 (8)	0.0472 (8)	0.0258 (6)	−0.0077 (6)	0.0000 (6)	0.0032 (6)
N2	0.0851 (12)	0.0559 (10)	0.0336 (8)	−0.0222 (9)	−0.0027 (7)	0.0072 (7)
N3	0.0823 (12)	0.0577 (10)	0.0342 (8)	−0.0247 (9)	−0.0032 (7)	0.0043 (7)
N4	0.0487 (11)	0.1340 (19)	0.0519 (10)	0.0007 (11)	0.0008 (8)	0.0018 (11)
N5	0.0481 (8)	0.0656 (10)	0.0309 (7)	0.0008 (7)	0.0053 (6)	0.0032 (7)
N6	0.0632 (10)	0.1060 (15)	0.0311 (8)	0.0225 (10)	−0.0005 (7)	0.0001 (8)

### Geometric parameters (Å, °)

**Table:**

C1—C6	1.383 (3)	C13—C14	1.407 (2)
C1—C2	1.385 (3)	C13—C17	1.437 (2)
C1—Cl1	1.722 (2)	C14—C15	1.389 (2)
C2—C3	1.367 (3)	C14—C18	1.482 (2)
C2—H2	0.9300	C15—H15	0.9300
C3—C4	1.364 (3)	C16—N4	1.141 (2)
C3—H3	0.9300	C17—N6	1.133 (2)
C4—C5	1.379 (3)	C18—C19	1.389 (2)
C4—H4	0.9300	C18—C23	1.390 (2)
C5—C6	1.379 (3)	C19—C20	1.376 (3)
C5—H5	0.9300	C19—H19	0.9300
C6—N1	1.430 (2)	C20—C21	1.382 (3)
C7—N1	1.350 (2)	C20—H20	0.9300
C7—C8	1.367 (2)	C21—C22	1.383 (3)
C7—C9	1.476 (2)	C21—C24	1.509 (3)
C8—N3	1.361 (2)	C22—C23	1.379 (3)
C8—C10	1.476 (2)	C22—H22	0.9300
C9—H9A	0.9600	C23—H23	0.9300
C9—H9B	0.9600	C24—H24A	0.9600
C9—H9C	0.9600	C24—H24B	0.9600
C10—C15	1.381 (2)	C24—H24C	0.9600
C10—C11	1.402 (2)	N1—N2	1.355 (2)
C11—C12	1.409 (2)	N2—N3	1.305 (2)
C11—C16	1.432 (3)	N5—H5A	0.8600
C12—N5	1.361 (2)	N5—H5B	0.8600
C12—C13	1.409 (2)		
			
C6—C1—C2	119.26 (18)	C12—C13—C17	116.25 (14)
C6—C1—Cl1	120.60 (14)	C15—C14—C13	118.14 (15)
C2—C1—Cl1	120.13 (16)	C15—C14—C18	119.53 (15)
C3—C2—C1	119.63 (19)	C13—C14—C18	122.32 (14)
C3—C2—H2	120.2	C10—C15—C14	121.52 (15)
C1—C2—H2	120.2	C10—C15—H15	119.2
C4—C3—C2	121.33 (17)	C14—C15—H15	119.2
C4—C3—H3	119.3	N4—C16—C11	177.9 (2)
C2—C3—H3	119.3	N6—C17—C13	175.17 (19)
C3—C4—C5	119.7 (2)	C19—C18—C23	117.71 (16)
C3—C4—H4	120.1	C19—C18—C14	121.75 (15)
C5—C4—H4	120.1	C23—C18—C14	120.54 (15)
C4—C5—C6	119.60 (19)	C20—C19—C18	120.83 (17)
C4—C5—H5	120.2	C20—C19—H19	119.6
C6—C5—H5	120.2	C18—C19—H19	119.6
C5—C6—C1	120.46 (15)	C19—C20—C21	121.72 (18)
C5—C6—N1	119.25 (16)	C19—C20—H20	119.1
C1—C6—N1	120.25 (16)	C21—C20—H20	119.1
N1—C7—C8	103.36 (15)	C20—C21—C22	117.39 (17)
N1—C7—C9	123.05 (14)	C20—C21—C24	120.83 (19)
C8—C7—C9	133.55 (15)	C22—C21—C24	121.78 (18)
N3—C8—C7	109.39 (14)	C23—C22—C21	121.56 (17)
N3—C8—C10	119.94 (14)	C23—C22—H22	119.2
C7—C8—C10	130.65 (16)	C21—C22—H22	119.2
C7—C9—H9A	109.5	C22—C23—C18	120.77 (17)
C7—C9—H9B	109.5	C22—C23—H23	119.6
H9A—C9—H9B	109.5	C18—C23—H23	119.6
C7—C9—H9C	109.5	C21—C24—H24A	109.5
H9A—C9—H9C	109.5	C21—C24—H24B	109.5
H9B—C9—H9C	109.5	H24A—C24—H24B	109.5
C15—C10—C11	120.01 (14)	C21—C24—H24C	109.5
C15—C10—C8	119.22 (15)	H24A—C24—H24C	109.5
C11—C10—C8	120.74 (15)	H24B—C24—H24C	109.5
C10—C11—C12	120.70 (15)	C7—N1—N2	111.79 (13)
C10—C11—C16	121.03 (15)	C7—N1—C6	128.73 (14)
C12—C11—C16	118.08 (15)	N2—N1—C6	119.48 (13)
N5—C12—C11	121.04 (15)	N3—N2—N1	106.45 (13)
N5—C12—C13	121.43 (14)	N2—N3—C8	109.01 (14)
C11—C12—C13	117.52 (14)	C12—N5—H5A	120.0
C14—C13—C12	122.10 (14)	C12—N5—H5B	120.0
C14—C13—C17	121.62 (15)	H5A—N5—H5B	120.0
			
C6—C1—C2—C3	−0.4 (3)	C12—C13—C14—C18	−177.47 (15)
Cl1—C1—C2—C3	−179.31 (16)	C17—C13—C14—C18	0.7 (3)
C1—C2—C3—C4	0.2 (3)	C11—C10—C15—C14	−0.6 (3)
C2—C3—C4—C5	0.0 (3)	C8—C10—C15—C14	177.05 (16)
C3—C4—C5—C6	−0.1 (3)	C13—C14—C15—C10	−0.1 (3)
C4—C5—C6—C1	−0.1 (3)	C18—C14—C15—C10	178.52 (16)
C4—C5—C6—N1	177.72 (17)	C15—C14—C18—C19	135.56 (19)
C2—C1—C6—C5	0.4 (3)	C13—C14—C18—C19	−45.9 (3)
Cl1—C1—C6—C5	179.25 (14)	C15—C14—C18—C23	−44.6 (2)
C2—C1—C6—N1	−177.45 (16)	C13—C14—C18—C23	133.90 (18)
Cl1—C1—C6—N1	1.4 (2)	C23—C18—C19—C20	0.9 (3)
N1—C7—C8—N3	−0.5 (2)	C14—C18—C19—C20	−179.29 (18)
C9—C7—C8—N3	177.18 (19)	C18—C19—C20—C21	−0.8 (3)
N1—C7—C8—C10	−178.75 (18)	C19—C20—C21—C22	−0.5 (3)
C9—C7—C8—C10	−1.1 (3)	C19—C20—C21—C24	179.2 (2)
N3—C8—C10—C15	−51.5 (2)	C20—C21—C22—C23	1.7 (3)
C7—C8—C10—C15	126.6 (2)	C24—C21—C22—C23	−177.98 (19)
N3—C8—C10—C11	126.11 (19)	C21—C22—C23—C18	−1.6 (3)
C7—C8—C10—C11	−55.7 (3)	C19—C18—C23—C22	0.3 (3)
C15—C10—C11—C12	0.3 (3)	C14—C18—C23—C22	−179.56 (16)
C8—C10—C11—C12	−177.31 (15)	C8—C7—N1—N2	0.0 (2)
C15—C10—C11—C16	175.27 (17)	C9—C7—N1—N2	−177.99 (17)
C8—C10—C11—C16	−2.4 (3)	C8—C7—N1—C6	179.69 (17)
C10—C11—C12—N5	−178.06 (16)	C9—C7—N1—C6	1.7 (3)
C16—C11—C12—N5	6.8 (2)	C5—C6—N1—C7	93.1 (2)
C10—C11—C12—C13	0.6 (2)	C1—C6—N1—C7	−89.1 (2)
C16—C11—C12—C13	−174.46 (16)	C5—C6—N1—N2	−87.3 (2)
N5—C12—C13—C14	177.34 (16)	C1—C6—N1—N2	90.6 (2)
C11—C12—C13—C14	−1.3 (2)	C7—N1—N2—N3	0.5 (2)
N5—C12—C13—C17	−0.9 (2)	C6—N1—N2—N3	−179.23 (16)
C11—C12—C13—C17	−179.63 (15)	N1—N2—N3—C8	−0.8 (2)
C12—C13—C14—C15	1.1 (3)	C7—C8—N3—N2	0.8 (2)
C17—C13—C14—C15	179.27 (16)	C10—C8—N3—N2	179.31 (17)

### Hydrogen-bond geometry (Å, °)

**Table:**

*D*—H···*A*	*D*—H	H···*A*	*D*···*A*	*D*—H···*A*
N5—H5B···N6^i^	0.86	2.56	3.221 (2)	134
N5—H5A···N4	0.86	2.91	3.540 (2)	125

Symmetry codes: (i) −*x*+1, −*y*+2, −*z*+1.
